# Integral feedback control is at the core of task allocation and resilience of insect societies

**DOI:** 10.1073/pnas.1807684115

**Published:** 2018-12-10

**Authors:** Thomas Schmickl, Istvan Karsai

**Affiliations:** ^a^Artificial Life Laboratory, Department of Zoology, Institute of Biology, Karl-Franzens-University Graz, A-8010 Graz, Austria;; ^b^Department of Biological Sciences, East Tennessee State University, Johnson City, TN 37614

**Keywords:** common stomach, swarm intelligence, collective behavior, homeostasis, self-regulation

## Abstract

Homeostatic self-regulation is a fundamental aspect of open dissipative systems. Integral feedback has been found to be important for homeostatic control on both the cellular and molecular levels of biological organization and in engineered systems. Analyzing the task allocation mechanisms of three insect societies, we identified a model of integral control residing at colony level. We characterized a general functional core mechanism, called the “common stomach,” where a crucial shared substance for colony function self-regulates its own quantity via reallocating the colony’s workforce, which collects and uses this substance. The central component in a redundant feedback network is the saturation level of this substance in the colony. An interaction network of positive and negative feedback loops ensures the homeostatic state of this substance and the workforce involved in processing this substance. Extensive sensitivity and stability analyses of the core model revealed that the system is very resilient against perturbations and compensates for specific types of stress that real colonies face in their ecosystems. The core regulation system is highly scalable, and due to its buffer function, it can filter noise and find a new equilibrium quickly after environmental (supply) or colony-state (demand) changes. The common stomach regulation system is an example of convergent evolution among the three different societies, and we predict that similar integral control regulation mechanisms have evolved frequently within natural complex systems.

## Significance

A key problem for complex systems is achieving resilience through their (often nonlinear) interactions between components. Social insect colonies are natural examples of highly scalable systems that achieve homeostatic self-regulation based on local interactions. We describe a “functional core model” that we identified in three different insect societies (wasps, ants, and honey bees). This core model is based on self-regulation through a shared (limited) substance that works as an information center and as a buffer system simultaneously. This system has several adaptive properties, as it is robust against environmental disturbances and insensitive against parameter changes. Finding such a “common core model” is of high significance in understanding the discrete transitions from individuality to sociality in several animal species through convergent evolution.

Homeostasis signifies the ability of a system to regulate its internal state in the face of changing external inputs (1). Although the term was originally used to refer to processes within living organisms, engineering systems have recently begun to have biological levels of complexity. Analyses of both biological and physical systems show that protocols and regulatory feedback loops that ensure optimality and robustness are the most important components to biological complexity (2–4). In biological systems, we can expect that successful protocols become highly conserved (and thus general) because they facilitate evolution and are difficult to change.

Integral feedback is used universally in engineering, and is likely to be ubiquitous in biology as well as in achieving homeostatic regulation or even “perfect adaptation” (3, 5). Integral feedback control is a fundamental engineering strategy for ensuring that the output of a system robustly sets an equilibrium value that is resilient to noise or variation in system parameters. Analyzing challenges and constraints that arise in efforts to engineer biological integral feedback controllers has shown that resource limitations that restrict the extent to which gains can be increased, along with other physical constraints that affect the feedback design, are crucial in understanding a system’s function (5–7). Yi et al. (5) argued that robust asymptotic tracking requires some kind of integral feedback as a structural property of the system. More generally, integral control may underlie the robustness of many homeostatic mechanisms. Saturation acting as a control for integral feedback is a key property for many negative feedback mechanisms (6). Recognizing the integral feedback control paired with proportional control mechanisms is important to biologists, because these could provide a mechanistic explanation of many biological phenomena.

The ability of a system to adapt primarily stems from the network connectivity that arises between the components of the system, without requiring specific fine-tuning of parameters (8–10). Ma et al. (11) computationally investigated all possible three-node network topologies to identify those that could perform adaptation. Only two major topologies emerged as robust solutions: a negative feedback loop with a buffering node and an incoherent feed-forward loop with a “proportioner node.” Minimal interaction networks containing these topologies are, within proper regions of parameter space, sufficient to achieve adaptation. More complex networks that robustly perform adaptation all contain at least one of these topologies at their core. Ma et al. (11) also found that negative feedback loops differ widely in their ability to facilitate adaptation. There is only one class of simple negative feedback loops that can robustly achieve adaptation: when the output node does not feed directly back to the input node but, instead, goes through an intermediate node that serves as a buffer.

Insect societies depend on coordinated complex infrastructure systems, such as supply chains, transportation and communication networks, and storage. Moreover, these systems have decentralized control, where individual insects make simple decisions based on local information (12–14). Even so, these societies show both high adaptability to changes and strong resilience against perturbations (15). Middleton and Latty (16) described how insect societies differ in their investing into three pathways to resilience: resistance, redirection, or reconstruction. The authors emphasized that the “resilient” system must return to a performance level that is equal to or exceeds performance predisturbance and also needs to stay functional.

We studied the task allocation mechanisms of wasps (17–20), ants (21), and honey bees (22, 23), and in all cases, we observed that workers were able to switch tasks without requiring noticeable changes in development or learning experience, or that these switches mostly caused short-term time delays (24). These switches result in rapid adjustment of the workforce, allowing the colony to compensate for disturbances. In all of the studied cases, we found a species-specific substance acting as the key for the regulation. These substances were stored in a saturable “common stomach” that acted as an information center and as a buffer. These common stomachs store and regulate inflow and outflow of substances, and the saturation of these substances in the common stomachs regulates the task allocation of material gatherers and users. In general, the local density of a substance (which represents the total saturation of the substance) is representative of the global system state if integrated over time. In this way, a simple cue-based regulation can suffice to regulate global system states without the need to develop a more complex signal- or language-based communication.

Our goal is to construct unified function-topology mapping that captures the essential topologies underlying the task allocation of these societies. We propose that the fundamental principle of the task allocation mechanism in insect societies is akin to integral control. We show that these systems are based on negative feedback, with the output indirectly fed back to the input through an intermediate node that also serves as a buffer. We will demonstrate that the core control system is resilient and able to regulate task allocation and substance flow to ensure a steady colony-level performance.

## Results

### Task Partition and Material Flow in Three Insect Societies.

**Fig. 1.**
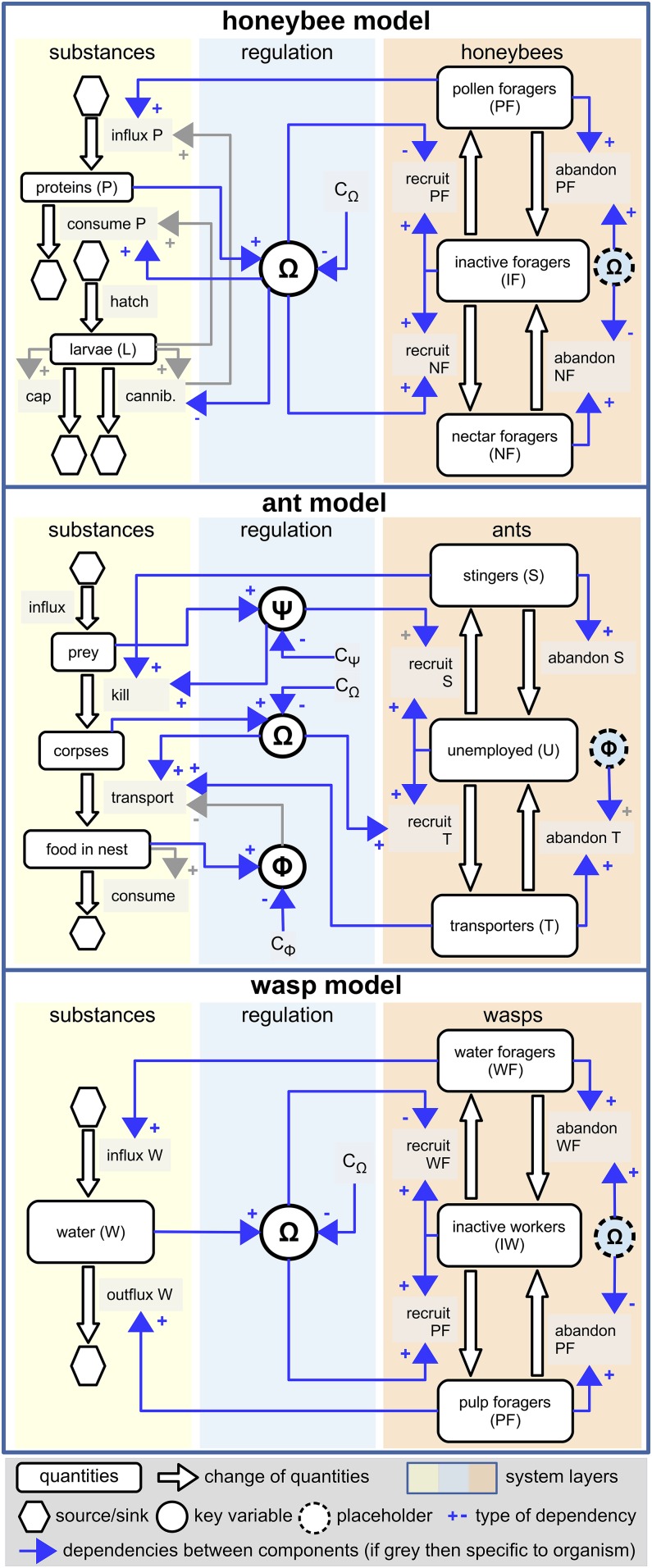
Schematic representations of the regulation systems of task allocation and material flows in three insect societies. The substance is shown in yellow, the workforce is shown in red, and the information layer is shown in blue. The regulating key variables in each insect society are stocks of shared substances (common stomach) and the saturation of those substances (Greek letters in circles). Recruitment and abandonment of individuals to and from tasks change accordingly with respect to the common stomach saturation. This, in turn, affects material flows, which determine the value of these key variables. A detailed model of each society is available in refs. 19, 21, and 23.

Many colony-level phenomena in insect societies require stable work performance, which ensures material flow, and a balanced workforce that handles those materials. We found that a control system we called the common stomach occurs in at least three major groups of eusocial insects (Fig. 1).

#### Wasps.

The nest construction behavior of *Polybia* sp. and *Metapolybia* sp. wasps relies on the saturation of water (needed for processing pulp) in the crops of water storers. Based on the common stomach’s water saturation level, water users and water collectors are recruited and their balance is maintained. This results in a steady nest construction that is resilient to perturbations (19).

#### Ants.

The hunting behavior of *Ectatomma ruidum* Roger depends on the density of living and dead prey animals in two distinct common stomachs. The saturation of these prey items in the hunting area and the nest regulates the numbers of stingers (killers) and transporters. This system ensures that if the prey is scarce, a generalist hunter will kill and transport the prey. If there is an abundance of prey, a balance between two specialized groups will emerge (as the hunting task is partitioned into stingers and transporters), which can better exploit the ephemeral bounty (21).

#### Bees.

Honey bees (*Apis mellifera* L.) collect pollen and nectar. The balance between nectar and pollen foragers is critical for colony growth and the success of overwintering (22). The balance between these forager groups is regulated by the protein saturation of worker bees that act as a common protein stomach. This regulation system can offset detrimental perturbation, such as rainy periods, pollen traps, or loss of foragers (23).

### The Common Regulatory Feedback Mechanism.

**Fig. 2.**
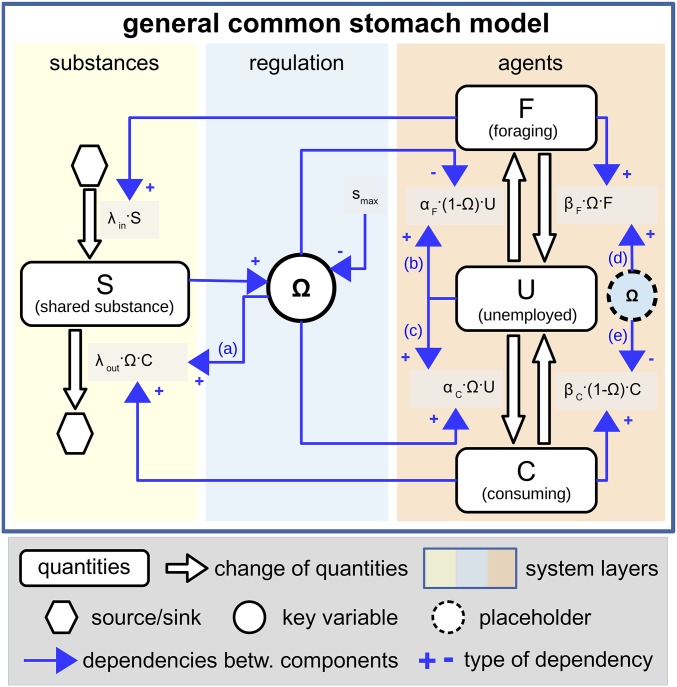
Schematic representation of the core regulatory mechanism found across the investigated insect societies. S is used for several important colony functions and is regulated by its own saturation level (Ω). Arrows with blue letters (a–e) indicate those causal connections where the common stomach saturation (Ω) affects the other components directly.

In each animal society that we studied, we observed at least one common core regulatory mechanism based on the saturation of a common stomach (Fig. 2). The common stomach is a saturable temporary storage of material residing at the colony level. In the case of wasps, it is the percentage of the current amount of water, which is available in the crops of a group of individuals that store water for water users and providers (25, 26). In general, the common stomach is a definite entity where a key substance accumulates and is accessible for both the foragers and the consumers. The common stomach has two main properties: It is a buffer, since it stores more material than is usually handled by a single individual, and it is also an information center, because the individuals are able to assess the quantity (and thus the saturation) of the material via specific interaction mechanisms.

The regulatory mechanism of the common stomach self-regulates the flow of material into the system, along with its usage (Fig. 2). When the saturation of the common stomach is low, more foragers are recruited from unemployed workers, while some of the consumers will become inactive (unemployed). This will result in an increased inflow and a reduced outflow of the substance in the colony, and the saturation level of the common stomach will increase. If the common stomach is highly saturated, it indicates a plenitude of material available to the colony, which will, in turn, elicit the recruitment of more consumers and the abandonment of foraging. This regulatory mechanism is akin to the integral feedback mechanisms described in many physical and biological systems at or below cellular levels. Here we identify and analyze a similar regulatory mechanism, one which works at the organizational level of animal societies. We stress that this is an important core mechanism that ensures the resilience and stability of insect societies.

### The Test of Stability of the Common Stomach Regulatory Feedback Core.

**Fig. 3.**
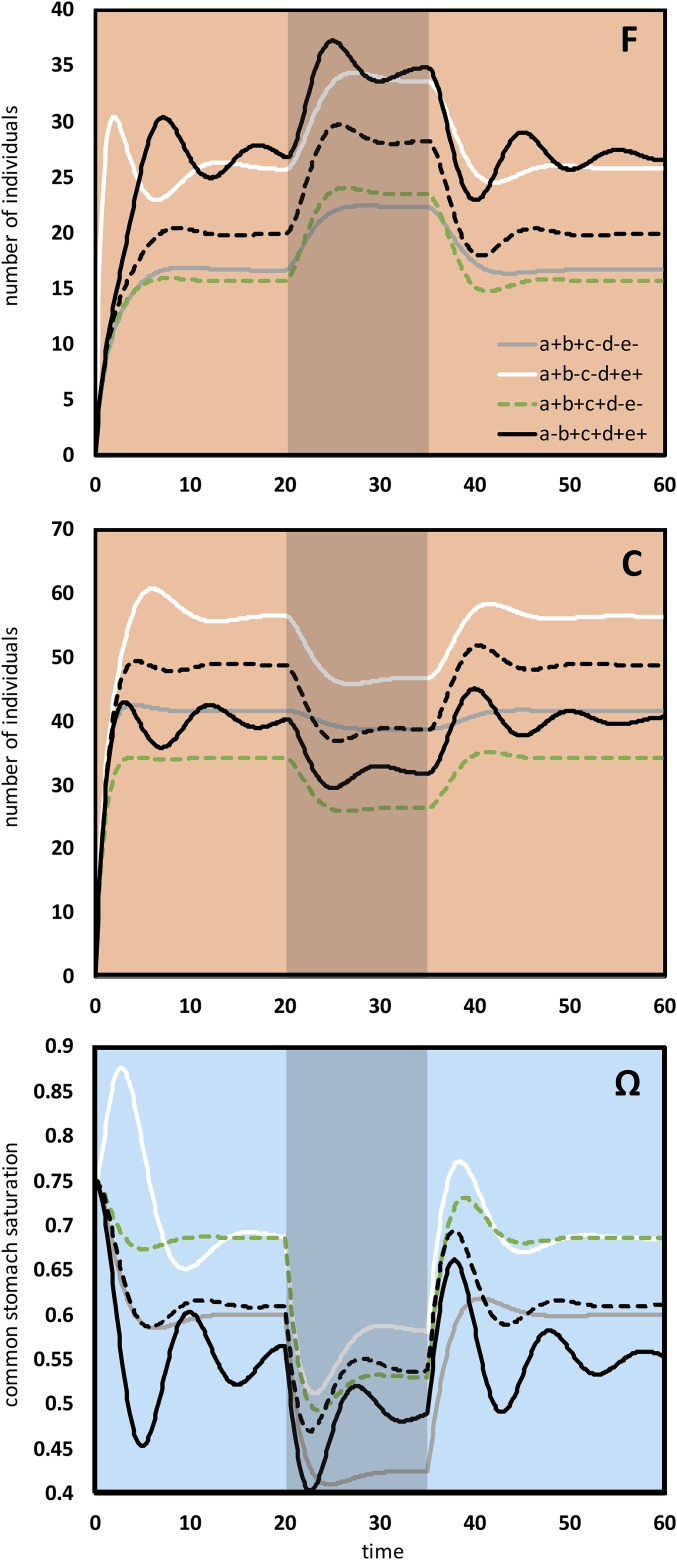
Dynamics of foragers (F), consumers (C), and common stomach saturation (Ω) after specific crucial feedback components (Fig. 2, arrows with letters a–e) are either turned on (+) or off (−). Between 20 and 35 units of time, a 20% leakage on the substance stock perturbed the system. A broken black line indicates a normal run with all feedbacks active, a black solid line indicates feedback to substance consumption cut, a white solid line indicates feedback to recruitments cut, a green broken line indicates feedback to abandonments cut, and a gray line indicates minimum required feedback.

The robustness of the system and its insensitivity to initial conditions both stem from 19 feedback loops (Fig. 2 and *SI Appendix*, Table S1), of which 13 are negative, thus stabilizing, and six are positive, thus escalating, feedback loops. The system shows a quick convergence to equilibrium, a robust counterbalancing of perturbations, and a fast return to the original equilibrium after perturbation (Fig. 3, black broken line). In our perturbation experiments, we experimentally induced a sudden leaking flow of the common stomach, and the colony compensated for this by recruiting more foragers and by laying off consumers. To assess the importance of each feedback loop, we systematically cut the links (Fig. 2, arrows with letters a–e) that connected the saturation of the common stomach (Ω) to other system components. The elimination of the feedback link to the outflow of the substance (Fig. 3, black line) resulted in increased fluctuations and stronger reactions to the perturbation. Eliminating the feedback link to the recruitment of both worker tasks had a similar, but weaker, effect (Fig. 3, white line). When the feedback link to the abandonment of both tasks was cut (Fig. 3, green broken line), the system reached a new equilibrium very fast, but it was less able to compensate for the effect of the perturbation. This is due to the lack of feedback, as consumers cannot abandon consumption while there is substance leakage. Thus, they decrease further the already scarce substance. After we also cut the feedback link to the recruitment of consumers, the system still functioned, but even less efficiently than before. We concluded that the minimum regulation of a working system occurs with feedback links of the common stomach saturation to substance outflow and to recruitment of foragers (Fig. 3, gray line).

**Fig. 4.**
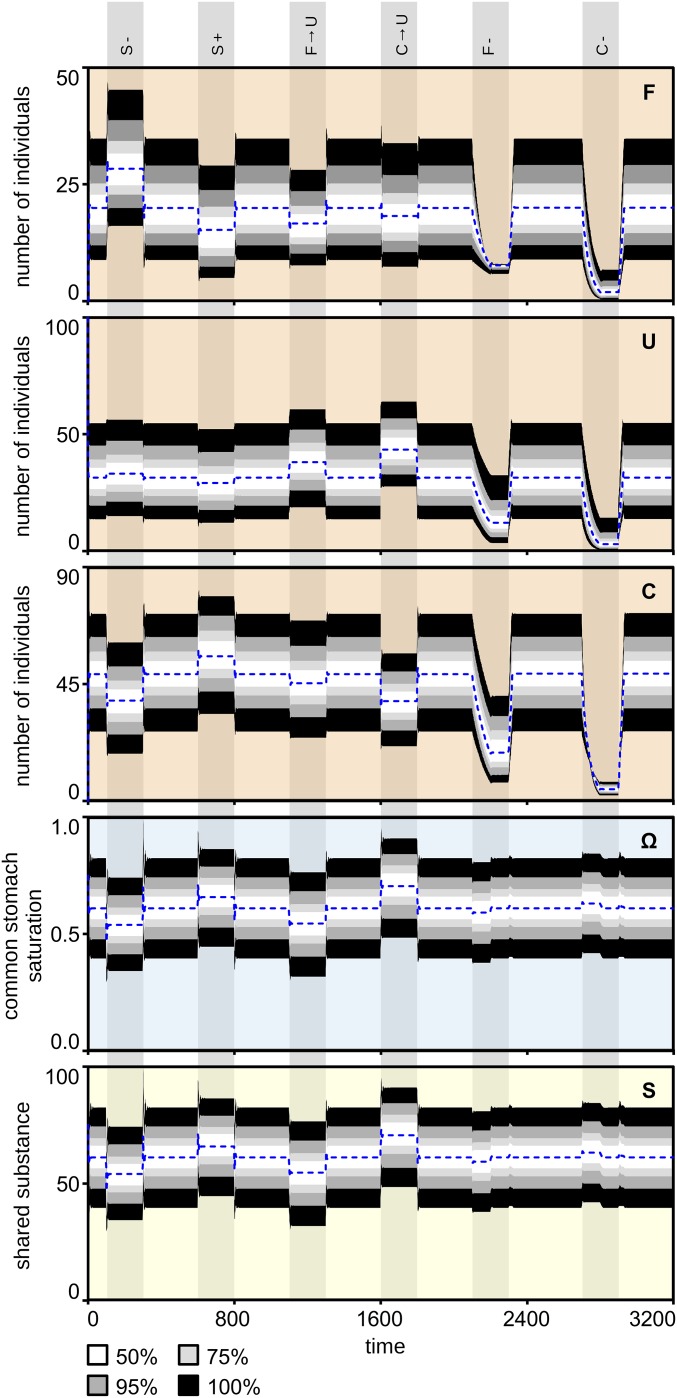
Sensitivity analysis of the model with a series of perturbation experiments. Main system variables are as follows: F, U, C, S, and Ω. Perturbations (vertical gray columns) are as follows: removing substance (S−), adding substance (S+), 25% of foragers turned to unemployed workers (F→U), consumers turned to unemployed workers (C→U), removal of foragers from the colony (F−), and removal of consumers from the colony (C−). A broken blue line indicates the median of the runs; a white ribbon indicates 50% of results; light and dark gray ribbons indicate 75% and 95% of the results, respectively; and a black ribbon indicates 100% of the results of 10,000 simulations.

There is heterogeneity in all organisms, even in insect societies, which are built from genetically closely related workers. Additionally, the environment and the state of the colony can influence various important life history parameters. To assess the effect of this diversity, targeted sensitivity analyses were carried out on different starting conditions (*SI Appendix*, Figs. S1 and S2). Pairing perturbation experiments with an extensive parameter sweep shows that the system is very robust and generally compensates for strong perturbations by keeping the amount of material (S) and the saturation of the common stomach (Ω) very stable, while counteracting the perturbations by rearranging the workforce [foragers (F), unemployed (U), and consumers (C)] (Fig. 4). After each perturbation, the system reestablished its equilibrium very quickly. Taking out (S−) or adding (S+) material to the substance stock was compensated for quickly via recruiting more foragers or consumers, respectively. Similarly, experimentally transferring individuals from one task group to another (F→U, C→U) was compensated for by the system recruiting heavily for the missing group or abandoning recruitment from the group with a surplus of workers. For example, when foragers were turned into unemployed workers, the number of consumers also decreased as relatively less substance was collected and the saturation of the common stomach decreased. This, in turn, made the abandonment of consumer tasks and recruitment of forager tasks higher. Removing foragers and consumers from the colony and then returning them to the colony only after the end of the perturbation (F−, C−) resulted in a decrease in the other task forces as well, while the level of substance and the saturation of the common stomach did not change significantly. For example, when foragers were removed, the substance influx decreased and the consumers abandoned their tasks, while unemployed workers were recruited mostly to perform the forager task. This robustness of the system was found to be insensitive to our key model parameters (*α*, *β*, and *λ* values) and also to the type of perturbations. Due to the buffering nature of the common stomach, it was able to absorb sudden changes and provide enough time for the colony to deploy compensatory measures, such as shifting the workforce accordingly (Fig. 4).

## Discussion

After analyzing the core of task regulation in wasp, bee, and ant societies, we found a common core mechanism among these regulations. Extracting and abstracting this core mechanism led us to the conclusion that the task regulation of insect societies can be considered akin to the integral control regulation already found in cellular and subcellular levels in biology. This has been predicted, but has not been found on higher organizational levels (6, 27). This regulation core, which we call common stomach regulation, ensures that the foraging and use of a crucial substance is regulated by the substance itself. The common stomach regulation is based on simple rules and local interactions, and is thus highly scalable, which is important in insect societies, where the colony size of one species can span from a few individuals to millions of individuals (28). The idea at the core of common stomach regulation seems simple: The material regulates itself by adjusting the workforce that handles that material. However, we found that this core has a high redundancy, which ensures reliability and robustness. A set of 13 negative feedback loops and six positive feedback loops ensures that the system reacts quickly to perturbations and is able to find a new equilibrium quickly. The common stomach saturation interacts at five different points (Fig. 2) in the system, and our analyses show that interacting only with the substance outflow and recruitment on one of the task groups would be sufficient for the system to work. However, this minimum configuration is less efficient than the fully connected network. For example, switching off the feedback from the common stomach to the abandonment of task-specific worker groups will achieve an equilibrium state more quickly, but the system is only able to compensate weakly if perturbed. Missing a feedback link from the common stomach state to its own outflow had the effect of delaying the onset of equilibrium, since these systems are oscillating more. We found that bees and ants have this kind of feedback in their substance regulations, but wasps might be lacking this feedback. Wasp societies are generally smaller and less homeostatic (more fluctuations can be observed) than larger bee and ant colonies. Their common stomach also contains water, which is probably a low-cost resource compared with the prey or pollen protein found in ants and bees. Task regulation in wasps happens via recruiting for and abandoning tasks that forage or use the substance of the common stomach. This indirect information can be assessed by the individuals through queuing delays (17, 18), which represent a simpler form of information collection than the mechanism found in bees and ants. The age and experience of individuals commonly influence individual task switches (29). However, on a shorter time scale, task allocation is balanced by quicker acting processes at the colony level. The inverse relationship between individual flexibility and colony size (17) allows insect societies to adapt on the fly via the task changing of individuals and/or recruitment of idle workers.

The assignment of individuals to different subtasks can be dynamic, which poses a decision-making problem for task switching (30). These individual and collective decisions are indicative of a computationally hard problem [NP-hard (nondeterministic polynomial-time hardness); not even easily “approximable”] (31), and are done either by a central task allocation agent (foreman) or, in the case of a distributed approach, by the individual worker itself (31). The core of the behavior regulation mechanism of insect societies has commonly been centered on fixed programs or threshold-based mechanisms (reviewed extensively in refs. 28, 32). These models are essentially based on sigmoid response curves, which ensure a nonlinear response to a linear stimulus. Paired with a positive feedback control, they are capable of simulating the emergence of division of labor from a principally homogeneous workforce. While these models commonly have high predictive value, their assumptions are sometimes less solid. The physical nature of the stimulus is often not specified, and is described instead by a global-state variable of the colony, such as “colony needs” or “food needed for the brood,” leaving it unclear how individual workers perceive those global states. The mechanisms of how these needs and stimuli are assessed and how outdated or wrong information regarding these needs can be avoided are often lacking. In several cases, the stimuli for task allocation were identified or predicted as a substance (33–35). We predict that many of the “operative”-state variables describing such stimuli will prove to be substances that can provide honest signals, due to physical conservation laws. This contrasts substance-mediated regulation to pure information exchange like “signaling,” “messaging,” or the bees’ dance language. Agrawal and Karsai (26) showed that linear interactions of individuals with the saturable common stomach, on average, would give a similar sigmoid stimulus response curve to that on which many models on division of labor operate. This means that although the real regulatory system operates on linear interaction patterns, an outside observer of the animals would see a sigmoid dependency of average animal behavior reacting to changing global colony conditions. The precision of assessing this signal often positively correlates with the frequency of interactions of the same individual with its nest mates (26, 36). Sigmoid response curves have been analyzed in comparison to common stomach regulation (21) on the hunting behavior of *E. ruidum*, where the model based on sigmoid curves failed the sensitivity test, while the common stomach model predicted feasible results even with strong perturbations.

Besides its role as an information center, the common stomach also provides a strong material buffer against fluctuations. The common stomach acts as an integrator and can reduce the impact of system noise (37) (*SI Appendix*, Fig. S3). Even if individual task switches have costs (delays), the system will still be very resilient to random noise and perturbations (24). Robust systems have the important advantage of having a larger parameter space in which they can evolve and adjust to environmental changes. The task allocation of wasp societies was modeled by using basic electric circuits (38), where electron flow stabilized the conversion of electrons to system heating via backpropagation of negative feedback mechanisms, while capacitors played the role of the buffering nature of the common stomach.

Csete and Doyle (3) emphasized that, particularly in biology, open-loop control does not necessarily provide satisfactory explanations. Closing control loops would provide better understanding of many biological processes. Yi et al. (5) also predict that more regulation mechanisms akin to integral feedback control will be discovered in biology. They provided several examples in complex manmade systems where these loops are used to engineer instruments and devices. In technical systems inspired by social animals, namely, swarm robotics and swarm intelligent systems, regulation through a shared limited resource was found to be beneficial. For example, robotic underwater swarms (39–41) can use shared resources to divide labor, multimodular robot interaction is inspired from resource distribution of substances (42), and hormone robotics (43) is based on a simulated substance flow within the body of the robot.

Similar networks that act as “computational devices” are responsible for many important cellular processes. These networks are very robust and have high adaptability (7). This ability to adapt stems primarily from the network connectivity, without requiring the fine-tuning of parameters (8–10). Identifying and understanding the nature of integral feedback control and similar control mechanisms are of fundamental importance for the understanding of biological regulation. Our findings support that the common stomach regulation system is an example of a closed-loop regulatory mechanism. The regulatory network reported here works on the supraindividual level in three different insect societies and is able to explain both the regulation of work and the resilience of these societies. Across three insect groups that evolved their eusociality separately from each other, the same general principle was “discovered” in biological evolution to achieve fast and stable regulation of task allocation. As each of these eusocial insect groups developed from nonsocial predecessor forms, the “common core” mechanism, which we described as the general model (Fig. 2), seems to be the product of convergent evolution. In all three cases, the same functional process has evolved but is expressed as a different physical manifestation dependent on the specific insect societies’ biology and ecology and their specific physics of interaction.

## Methods

### The Model.

The model was developed in Vensim (44) and is described in detail in *SI Appendix*.

The amount of substance S in the common stomach increases by the influx provided by the collectors and decreases by the outflow caused by the substance consumers:

$$\frac{dS}{dt}=\lambda_{in}F\left(t\right)-\lambda_{out}\text{Ω}\left(\text{t}\right)C\left(t\right),$$ [1]

where *λ*_*in*_ and *λ*_*out*_ are scaling constants for influx and outflux rates and *F* and *C* represent the numbers of foragers and consumers, respectively, while the saturation of the common stomach is Ω(t).

The recruitment of foragers is inversely proportional to the saturation of the common stomach, while the abandonment of the collector task is proportional to the saturation of the common stomach:

$$\frac{dF}{dt}=\alpha_{F}\left(1-\text{Ω}\left(\text{t}\right)\right)U\left(t\right)-\beta_{F}\text{Ω}\left(\text{t}\right)F\left(t\right),$$ [2]

where *α* and *β* are recruitment and abandonment rates and *U*(*t*) is the number of unemployed workers. The dynamics of consumers can be described similar to the dynamics of the foragers, but the recruitment is directly proportional to Ω(t), while the abandonment of the consumer task is inversely proportional to Ω(t):

$$\frac{dC}{dt}=\alpha_{C}\text{Ω}\left(\text{t}\right)U\left(t\right)-\beta_{C}\left(1-\text{Ω}\left(\text{t}\right)\right)C\left(t\right).$$ [3]

The specialized task groups are recruited from the unemployed workforce, and they also revert to unemployed after they abandon their specialized tasks.

### Perturbation Experiment and Sensitivity Analyses.

For all three focal insect species, we provided a detailed parameter sensitivity analysis (SA), as most model parameters were estimated from field experiments (19, 21, 22). SA is also crucial for the general core model we present here to ascertain that the stability of this system is emerging from the proposed control mechanism. We used the SA tool of Vensim (44) for running 10,000 independent experiments with randomized parameter sets (Latin hypercube sampling). All parameters (*α*, *β*, and *λ* values) were drawn from a uniform random distribution between 0.25 and 0.75, except *λ*_*in*_, which was chosen to be between 0.5 and 1.0. In addition, a series of extra perturbation experiments was carried out (Table 1).

**Table 1.** Perturbations during sensitivity analysis

Perturbation type	Perturbation phase (units of time)	Affected stock	How affected (in every unit of time)
S−	100–300	S	Reduced by 20%
S+	600–800	S	Added 7 units
F→U	1,100–1,300	F, U	25% of F moved to U
C→U	1,600–1,300	C, U	25% of C moved to U
F−	2,100–2,200	F	Reduced by 5%
C−	2,700–2,800	C	Reduced by 5%

In the remaining time intervals (before, after, and between the perturbations), the model was running with the standard values and all removed individuals (after the fifth and sixth experiments) were returned into the colony at a rate of 1.0 unemployed individuals per time step.

## Supplementary Material

Supplementary File
