# Supplementary material for: Integral feedback control is at the core of task allocation and resilience of insect societies
Source: Proc Natl Acad Sci U S A. 2018 Dec 10;115(52):13180–5. doi: 10.1073/pnas.1807684115 (PMC6310805; doi:10.1073/pnas.1807684115)
Supplement: Supplementary File [file pnas.1807684115.sapp.pdf]

Supporting Information (SI) Appendix

Thomas Schmickl and Istvan Karsai.

Integral feedback control is at the core of task allocation and resilience of insect societies

Table of Contents

A. Dependency of equilibria on starting conditions of key system variables
B. Dependency of equilibria on substance influx and outflux rates
C. Effect of zero-mean noise on the predicted dynamic equilibria
D. Assumptions and fundamental concepts of the model
E. Identification and classification of feedback loops in the common stomach system
F. References

A. Dependency of predicted equilibria on starting conditions

In the main body of the paper, we provided detailed sensitivity analyses on the system's dependence on the different combinations of varying initial values of all parameters (Fig. 4, Table 1). To further investigate the dependency of the emergent equilibria on initial conditions, we performed a set of targeted simulation experiments where a single variable's initial value was varied in each simulation run. The main system variables converge to the same dynamic equilibria regardless of the starting conditions of these system variables (Fig. S1).

www.pnas.org/cgi/doi/10.1073/pnas.1807684115

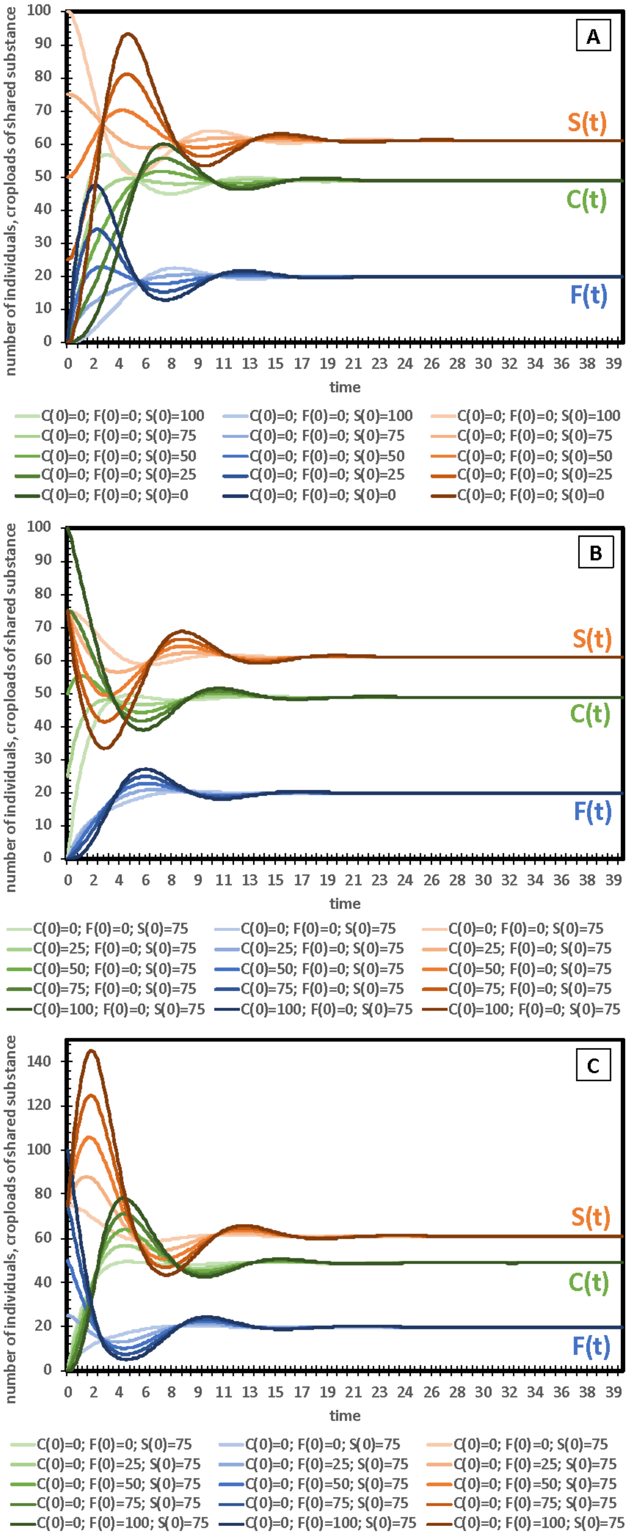

Figure S1. Dependence of equilibria for the system variables S(t), C(t) and F(t) in the general model (Fig. 2), for the starting value of S(0): A, C(0): B, and F(0): C. All other parameter values were kept at the standard values (Δt = 0.125, 4th order Runge-Kutta numerical solver).

B. Dependency of predicted equilibria on substance influx and outflux rates

In uncontrolled natural environments, the flux rates of substances in and out of the common stomachs are strongly dependent upon the focal animal species, the state and the age of the colonies, as well as on frequently changing environmental conditions. We performed a set of targeted simulation experiments in which one of those flux rates was varied to investigate how the predicted dynamic equilibria are affected by varying the rates of the substance inflows and outflows. In all the analyzed cases, the main system variables quickly converge to dynamic equilibria, though the levels of these equilibria depend on the flux rates (Fig. S2). These results suggest that the systems are able to adapt to colonial and environmental changes by rearranging their workforce to reach steady substance processing.

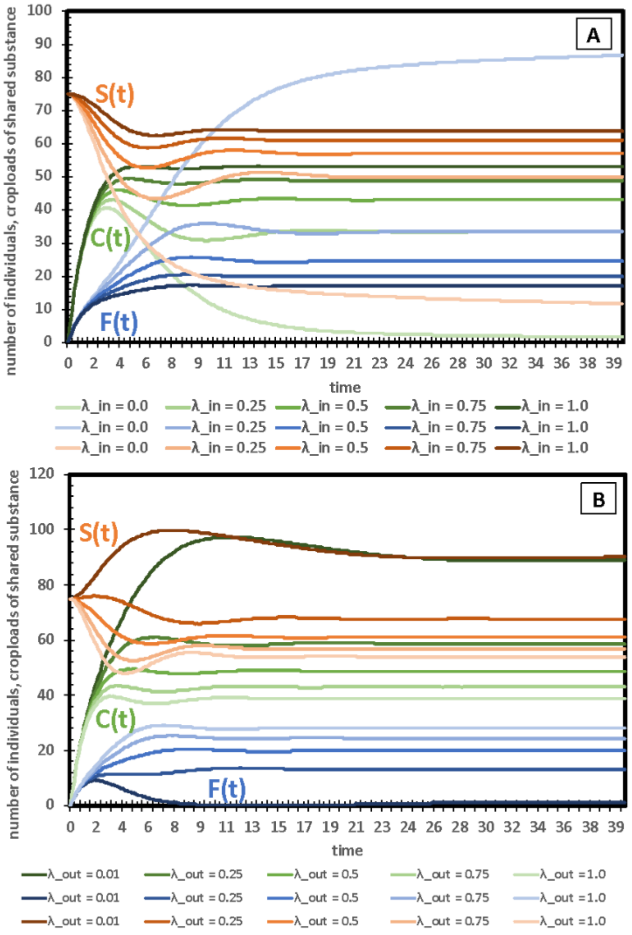

Figure S2. Dependence of equilibria for the system variables S(t), C(t), and F(t) on **A**: influx rate (λ_{in}) and **B**: outflux rate (λ_{out}) of the substance into and out of the common stomach (Fig. 2). All other parameter values were kept as the standard values ($\Delta t = 0.125$, 4th order Runge-Kutta numerical solver).

C. Effect of zero-mean noise on the predicted dynamic equilibria

The environmental influx of the shared substance, along with the colony's internal usage of the substance, can vary throughout the day (1, 2). Thus, it is important to investigate the stability of the system with respect to noise. Variations in the influx may stem from the stochasticity of substance retrieval (scouting success in honeybees (3). Crop-loads returned by foragers vary individually and depend on environmental factors (4, 5), while harvesting success in ants may vary with environmental food density (6, 7).

To investigate how the predicted dynamic equilibria are affected by varying levels of noise on substance inflows and outflows, we performed a set of targeted simulation experiments where one of the flux rates was additively transformed by normal distributed zero-mean noise (non-proportional transformation). In each time step, a randomly (normal distribution) generated value (between zero and the noise level {0.0, 0.25, 0.5, 0.75} and a standard deviation of 66.6% of the noise level) was added or subtracted to the influx or outflux value. We analyzed the response of the system through the behavior of its main system parameters (Fig. S3). The model is stable against high levels of zero-mean noise, and the value of each main system parameter remains close to its equilibrium. The fluctuations are strongest in the shared substance depot S(t). However, this is expected, as the common stomach is the integrator of these flows, and this property of integration tends to decrease the impact of high frequency system noise (8). The common stomach also has a strong buffering effect, so with the additional effect of the regulating behavioral feedback loops, the compositions of worker forces are less affected by the noise.

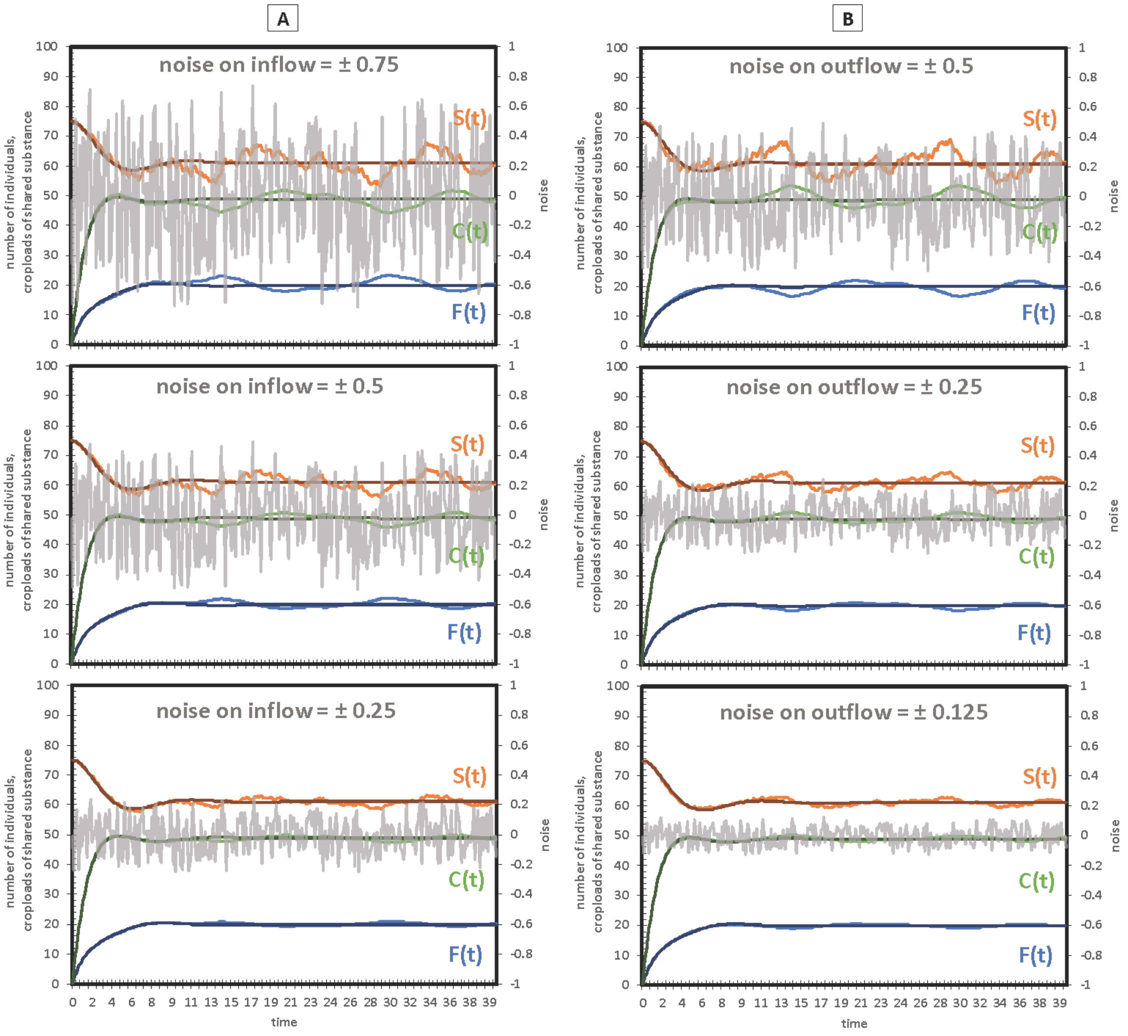

Figure S3. Effect of zero-mean noise (gray) in the substance inflow (**A**) and outflow (**B**) on the dynamic equilibria of the system variables S(t), C(t) and F(t) (Fig. 2). All other parameter values were kept at the standard values (Δt = 0.125, 4th order Runge-Kutta numerical solver).

D. Assumptions and fundamental concepts of the model

We built our general model as a system of coupled ordinary differential equations (ODEs) which we solve numerically by a 4th order Runge-Kutta method with a time step size of Δt = 0.125 time units. In the following, we detail the terms and parameters of the three main equations:

We assume that there are two major processes that determine the change of shared substance S(t) in the colony (equation 1):

$$\frac{dS}{dt} = \lambda_{in} F(t) - \lambda_{out} \Omega(\mathrm{t}) C(t) \qquad [1]$$

The first term models that every active forager F(t) transports an average volume of λ_{in} of the shared substance S(t) into the common stomach. We assume that the foragers will not always arrive with a full load, but we also assume that the foragers will give some significant contribution. Thus, we set our default value of λ_{in} to 0.75 volume units per worker per time step.

The second term describes the assumption that the substance consumed by the colony is proportional to the number of consumers C(t) and the common stomach saturation Ω(t). The second term represents an implementation of the mass-action law, since the consumption depends on both: on the relative abundance of the substance (Ω(t)), and on the available consumers that can use it. The term λ_{out} is a scaling parameter representing the ease of retrieving material from the common stomach. This value is close to its maximum (1.0) when the common stomach is full and close to its minimum (0.0) when the common stomach is empty. Therefore, we set the value of λ_{out} to 0.5 volume units per worker per time step.

The change in the number of foragers (equation 2) and consumers (equation 3) is determined by two processes: recruitment of unemployed workers to the special task, as well as abandonment of that special task and becoming unemployed

$$\frac{dF}{dt} = \alpha_F\big(1 - \Omega(\mathrm{t})\big)U(t) - \beta_F\Omega(\mathrm{t})F(t) \qquad [2]$$

$$\frac{dC}{dt} = \alpha_C\Omega(\mathrm{t})U(t) - \beta_C\big(1 - \Omega(\mathrm{t})\big)C(t) \qquad [3]$$

We assume that both the recruitment and the abandonment can also be expressed by a mass-action law where the number of the individuals of a task group is multiplied with the saturation of the common stomach (or with its inverse) and a scaling coefficient. The two coefficients (α and β) model the speed of these two processes. Similarly, as discussed for equation 1, these coefficients can change between 0.0 and 1.0 depending on the fullness of the common stomach. Assuming an average half-full common stomach (9), we set all recruitment and abandonment rates to 0.5. Whether or not this recruitment or the abandonment is proportional or inversely proportional to the common stomach is based on (10). When a substance is rare in a colony, then the common stomach saturation will be low. A low saturation of the common stomach promotes the recruitment of foragers and the abandonment of consumers. We express this as an inverse relationship (1 - Ω(t)), or the "degree of emptiness of the common stomach" in the multiplied terms.

We assume that workers distribute the shared substance amongst themselves via a process of worker-to-worker interactions that are similar to a diffusion process. This process has been described in detail in (9). As a consequence of these inter-individual substance exchanges in large colonies, individual differences in shared substance possession will "average out" over time. The saturation of the common stomach is expressed by $\Omega(t)=S(t)/s_{max}$, where s_{max} is the overall storage capacity of the common stomach. This maximum value depends on the number of individuals (N=100) which engage in the process of the shared substance (foragers, users and storers). We assumed that all individuals have a maximum capacity of crop volume (1.0) to store the substance, therefore the capacity of the substance in the colony is the product of these 2 parameters. For simplicity, we further assumed that the number of colony individuals stays constant during the studied time periods (several hours to several days): N = C(t)+U(t)+F(t). The rates of change of the system variable U(t) can thus be calculated directly from the

constant colony population size (N) and the values of the two system variables C(t) and (F(t). Consequently, we have conservation of mass in the model layer that tracks the workers (agents, right red-shaded column in Fig. 2), which represents a closed system. In contrast, the model layer that tracks substances (left yellow-shaded column in Fig. 2) is modeled as an open system, containing an explicit source (inflow) and an explicit sink (outflow) in the system. The middle layer (blue shaded area in Fig. 2) acts as a regulating mediator between these two subsystems.

E. Identification and classification of feedback loops in the common stomach system

The behavioral processes that are described by the terms of the RHS of equations 1-3 establish several positive and negative feedback loops that govern the system dynamics and the resilience and convergence properties of system variables. We identified 13 negative feedback loops and 6 positive feedback loops in our general model (Table S1):

Table S1. Feedback loops of the system.

Loop	Variables included in each feedback loop	Type	Effect
#1	S(t)→ Ω(t)→outflux(t)→S(t)	negative	Prevents CS from running empty
#2	U(t)→recruitment$_F$(t)→U(t)	negative	Balances the number of unemployed agents
#3	U(t)→recruitment$_C$(t)→U(t)	negative	Balances the number of unemployed agents
#4	F(t)→abandonment$_F$(t)→F(t)	negative	Balances the number of active foragers
#5	C(t)→abandonment$_C$(t)→C(t)	negative	Balances the number of active consumers
#6	S(t)→ Ω(t)→abandonment$_C$(t)→C(t)→outflux(t)→S(t)	negative	Prevents the CS from running empty
#7	S(t)→ Ω(t)→abandonment$_F$(t)→F(t)→influx(t)→S(t)	negative	Prevents the CS from overfilling
#8	S(t)→ Ω(t)→recruitment$_C$(t)→C(t)→outflux(t)→S(t)	negative	Prevents the CS from running empty
#9	S(t)→ Ω(t)→recruitment$_F$(t)→F(t)→influx (t)→S(t)	negative	Prevents the CS from overfilling
#10	U(t)→recruitment$_F$(t)→F(t)→abandonment$_F$(t)→U(t)	positive	Enhancing foraging
#11	U(t)→recruitment$_C$(t)→C(t)→abandonment$_C$(t)→U(t)	positive	Enhancing consumption
#12	U(t)→recruitment$_F$(t)→F(t)→influx(t)→S(t)→ Ω(t)→recruitment$_C$(t)→U(t)	negative	Balancing the tasks
#13	U(t)→recruitment$_C$(t)→C(t)→outflux(t)→S(t)→ Ω(t) →abandonment$_C$(t)→U(t)	positive	Enhancing consumption
#14	U(t)→recruitment$_C$(t)→C(t)→outflux(t)→S(t)→ Ω(t) →recruitement$_F$(t)→U(t)	negative	Balancing the tasks

#15	U(t)→recruitment$_F$(t)→F(t)→influx(t)→S(t)→ Ω(t) →abandonment$_F$(t)→U(t)	positive	Enhancing influx
#16	U(t)→recruitement$_C$(t)→C(t)→outflux(t)→S(t)→ Ω(t)→abandonment$_F$(t)→U(t)	negative	Regulating outflux
#17	U(t)→recruitment$_F$(t)→F(t)→influx(t)→S(t)→ Ω(t)→ abandonment$_C$(t)→U(t)	negative	Regulating influx
#18	U(t)→recruitment$_F$(t)→F(t)→influx(t)→S(t)→ Ω(t)→recruitment$_C$(t)→C(t)→abandonment$_C$(t)→U(t)	positive	Enhancing influx
#19	U(t)→recruitment$_C$(t)→C(t)→outflux(t)→S(t)→ Ω(t) →recruitment$_F$(t)→F(t)→abandonment$_F$(t)→U(t)	positive	Enhancing consumption

In this table, the variable influx(t) represents the positive term in the RHS of equation 1, while the variable outflux(t) represents the negative term. The variables recruitment$_C$(t) and recruitment$_F$(t) represent the positive terms on the RHS of equations 2 and 3, while the variables abandonment$_C$(t) and abandonment$_F$(t) represent the negative terms.

F. References

1. Dussutour A, Beekman M, Nicolis SC, Meyer B. (2009) Noise improves collective decision-making by ants in dynamic environments. *P Roy Soc B-Biol Sci* 276:4353-4361. doi:10.1098/rspb.2009.1235.

2. Deneubourg JL, Pasteels JM, Verhaeghe JC. (1983) Probabilistic behaviour in ants: a strategy of errors? *J Theor Biol* 105:259–271. doi:10.1016/S0022-5193(83)80007-1.

3. Seeley TD (1983) Division of Labor between Scouts and Recruits in Honeybee Foraging. *Behav Ecol Sociobiol* 12:253–259.

4. Huang MH, Seeley TD (2003) Multiple unloadings by nectar foragers in honey bees: A matter of information improvement or crop fullness? *Insect Soc* 50:330-339. doi:10.1007/s00040-003-0682-4.

5. Li L, Peng H, Kurths J, Yang Y, Schellnhuber HJ (2014) Chaos–order transition in foraging behavior of ants *Proc Natl Acad Sci USA* 111:8392-8397. doi.org/10.1073/pnas.1407083111.

6. Campos D, Bartumeus F, Méndez V, Andrade JS, Espadaler X (2016) Variability in individual activity bursts improves ant foraging success *J R Soc Interface* 20160856. doi: 10.1098/rsif.2016.0856.

7. Schmickl T, Karsai I (2014) Sting, carry and stock: How corpse availability can regulate de-centralized task allocation in a Ponerine ant colony. *PLoS ONE* 9(12):e114611.

8. Peng H, Li L, Yang Y, Wang C (2009) Parameter estimation of nonlinear dynamical systems based on integrator theory. *Chaos* 19:033130. Doi:10.1063/1.3216850

9. Agrawal D, Karsai I (2016) The mechanisms of water exchange: The regulatory roles of multiple interactions in social wasps. *PLoS ONE* 11(1):e0145560.

10. Karsai I, Wenzel JW (2000) Organization and regulation of nest construction behavior in Metapolybia wasps. *J Insect Behav* 13:111-140.
